# Development of L-carnosine functionalized iron oxide nanoparticles loaded with dexamethasone for simultaneous therapeutic potential of blood brain barrier crossing and ischemic stroke treatment

**DOI:** 10.1080/10717544.2021.1883158

**Published:** 2021-02-15

**Authors:** Xianfeng Lu, Yaohui Zhang, Lixiang Wang, Guichen Li, Jianyuan Gao, Ying Wang

**Affiliations:** a Department of Pediatrics, Shanxi Provincial People's Hospital, Taiyuan, China;; b Department of Neurology, Luoyang Central Hospital Affiliated to Zhengzhou University, Luoyang, P.R.China;; c Department of Neurology, Laigang Hospital Affiliated to Shandong First Medical University, Jinan, China; d Department of Clinical Psychology, Qingdao Mental Health Center Clinical Psychology, Qingdao, P.R. China; e Department of Geriatrics, Xijing Hospital, The Fourth Military Medical University, Xi’an, P.R. China; f Internal Medicine-Neurology, Liaocheng People's Hospital, Liaocheng, PR China

**Keywords:** Magnetic nanoparticles, L-carnosine, PLGA polymer, dexamethasone, ischemic stroke

## Abstract

The development of suitable drug delivery carriers is significant in biomedical applications to improve the therapeutic efficiency. Recent progress in nanotechnological fields, paved the way for the formulation of variety of drug carriers. The brain disorders such as ischemic stroke, brain cancer, and CNS disorders were poorly treated due to the presence of blood brain barrier that hinders the passage of drugs to the brain. Hence, the formulated drugs should have the ability to cross the blood-brain barrier (BBB) for ischemic stroke treatment. In the present work, we have synthesized PLGA functionalized magnetic Fe_3_O_4_ nanoparticle (MNP) with L-carnosine peptide (LMNP) composite loaded with dexamethasone (dm@LMNP) and demonstrated as efficient drug delivery platform for simultaneous BBB crossing and treatment of ischemic stroke. The surface morphology, particles size and zeta potential of the prepared material was studied from SEM, PSD, PDI and TEM analyses. The drug loading of dexamethasone in LMNP (dm@LMNP) vesicles was found to be 95.6 ± 0.2%. The *in vitro* drug release kinetics displayed that prepared composited LMNP material provides controlled and sustainable releasing efficiency at pH 7.4 and 5.8 when compared to the PLGA NPs and free dexamethasone drug molecules. The cytotoxicity and the biocompatibility test results were found to be satisfactory. The L-carnosine loaded nano-formulation has been greatly leads to effective BBB crossing to access the brain tissues. These results showed that the Fe_3_O_4_ nanoparticles/PLGA polymer can be used as an effective drug carrier for the treatment of stroke and simultaneous blood brain barrier crossing.

## 1. Introduction

Stroke can be defined as an acute neurological dysfunction caused by the interruption to the vasculature supplying the brain (Rhim et al., 2013; Sacco et al., 2013; Kyle & Saha, 2014). Stroke is the fifth leading cause of death and most common cause for disability and approximately 80,000 people were affected every year worldwide growing continuously due to the aging of population (Han et al., 2016). At present, thrombolytic agents, neuro therapeutic agents and recombinant tissue plasminogen activator (rTPA) are approved by FDA for the treatment of stroke in which the clot gets dissolved and blood flow was restored (Jiang et al., 2018; Bao et al., 2018). However, very few stroke patients were benefited with the rTPA because of its narrow therapeutic window which is less than 4.5 h and the safety issues including neurotoxicity and intracerebral bleeding (Yang et al., 2019). The brain did not have enough blood supply by a bleeding vessel leads to hemorrhagic stroke or blockage of a blood vessel due to the blood clot can cause ischemic stroke and 87% of stroke is ischemic (Thompson & Ronaldson, 2014). The cost estimation of stroke can have a socio economic impact and it may be a personal burden. According to the American Heart association, in 2008, the total cost of stroke was estimated as 34.3 billion dollars and 8 billion per year based on the report of the stroke association in England alone respectively (Kyle & Saha, 2014). In our human body, central nervous system is the most critical and sensitive system and the efficient function of CNS needs regulated extracellular environment to maintain the concentration of K^+^, Na^+^ and Ca^2+^ ions within narrow ranges (Hawkins & Davis, 2005). The essential interface between the CNS and periphery is called blood brain barrier mainly constituted by endothelial cells connected by tight junctions and adherens junctions and a sparse layers of pericytes (Thomsen et al., 2013; Mc Carthy et al., 2015; Saraiva et al., 2016). Blood brain barrier is a physical, metabolic and transport barrier which controls the transfer of substances from blood to neuro tissues and vice versa (Rhim et al., 2013; Oller-Salvia et al., 2016). The blood brain barrier was first put forward by Goldman based on the previous hypothesis by Lewandowsky which states that there is a barrier between blood and barrier prevents the transfer of dyes (Jiang et al., 2018). The function of BBB includes maintaining the ion balance, nutrient transporter and the barrier to protect the brain pathogens (Patel & Patel, 2017). However, the BBB is the blocking barrier for neuro pharmaceuticals drugs because 100% of large molecule drugs and 98% of small drugs cannot penetrate through the barrier (Pardridge, 2007; Jiang, 2013; Kreuter, 2014; Xie et al., 2019). The partial disruption of BBB leads to stroke which is evident in 33% of ischemic human patients. If the BBB gets disrupted, the ischemia was observed in 4 to 5 h in experimental rats and 12.9 h in humans (Han et al., 2016). In addition to that, the degree of leakage is not sufficient for delivery of important drugs for effective ischemic treatment and the BBB crossing was confronted as big challenge in drug delivery.

The development of drug delivery systems for the treatment of brain diseases received paramount interest in recent decades. Brain diseases are poorly treated and have to grow in an aggressive manner in the future as the population of CNS patients and brain disorders are increasing (Dong, 2018). Recent progress in nanotechnological fields paved the way for the design strategies and synthesis of novel nanostructures for biomedical applications. Nanotechnology also provides further insight in the accurate control over drug-cellular interactions and in vitro prediction of cell responses in CNS based on their stimulation to molecular characteristics. Nanoparticles based drug formulations has been significantly developed for the effective delivery of drugs across the BBB barrier for the treatment of brain disorders such as stroke, Alzheimer’s, Parkinson etc. There are lot of nanomaterials have been investigated for targeted drug delivery and BBB crossing such as inorganic nanoparticles (Qiao et al., 2012; Yim et al., 2012; Frigell et al., 2014), polymeric nanoparticles (Patel et al., 2012), liposomes (Chen et al., 2010), micelles (Liu et al., 2008), etc because of their high drug loading capacity, low toxicity and biocompatibility. Magnetic iron oxide nanoparticles (MN) have important characteristics which make them attractive candidate in variety of biomedical applications include cell separation and detection, contrast agents in MRI, drug delivery etc. In particular iron oxides are highly stable in physical and chemical conditions, environmental benign and biocompatible. Moreover, Fe_3_O_4_ magnetic nanoparticles have been widely used as the drug carrier owing to its super magnetic behavior, and biocompatibility (Majeed et al., 2013; Shagholani et al., 2015; Jahangirian et al., 2017; Mcnamara et al., 2017). Nanoparticles encapsulated with the polymers like PEG, PLGA, Chitosan are expected to enhance the stability of the colloidal solution resulted in effective BBB crossing and enable the drug delivery to CNS (Saeedi et al., 2019). Moreover, the elimination of drugs could be protected using this semi permeable membrane of synthetic polymer, nanofibers etc. PLGA is considered as one of the extensively studied polymeric nanoparticles to carry drugs to the targets especially in brain disorders (Grumezescu et al., 2019) due to its biocompatibility, biodegradability, physiochemical versatility and so on (Ficai et al., 2018). For instance, curcumin loaded in PLGA nanoparticles modified with g7 ligand is efficient in BBB crossing (Barbara et al., 2017). Dexamethasone is a glucocorticoid drug possess anti-inflammatory properties have been used as potential candidate for the treatment of inflammatory diseases like stroke (Komane et al., 2018). In this work, Fe_3_O_4_ nanoparticles/PLGA polymer loaded dexamethasone functionalized with L- carnosine was prepared and investigated for the targeted drug delivery for the treatment of ischemic stroke and the simultaneous blood brain barrier crossing.

## 2. Materials and methods

### 2.1. Chemicals

All chemicals were of analytical grade and used without further purification. All the solutions were prepared with millipore water with electrical resistivity of 18MΩ-cm produced by an ultrapure water system. Dexamethasone was purchased from Haidebei Ocean Biochemical Co. Ltd, Jinan, China. PLGA (MW ~ 10,000 g/mol; lactic acid and glycolic acid ratio was 75/25) was purchased from Sinopharm chemical reagent Co. Ltd, Shanghai, China. Iron chloride salts and urea were brought from Sigma Aldrich.

### 2.2. Characterization

The XRD diffractograms were taken from Pan analytical X-Ray diffractometer with scanning range of 20 to 80 degree with scanning speed of 48 per min, using Cu Ka radiation. The surface morphology and the crystallite size were obtained from Scanning Electron Microscope (SEM) HITACHI S–2400, Japan and Transmission Electron Microscopy (TEM), H7500, Hitachi Ltd., Japan at 120 kV respectively. FTIR spectra were recorded in the range of 2000–1200 cm^2^ using Nicolet-170 SX FT-IR spectrophotometer. The hydrodynamic size and the surface charge were obtained at room temperature from dynamic light scattering measurements using Malvern-DTS Ver 4.20.

### 2.3. Preparation of iron oxide nanoparticles

Iron oxide nanoparticles are prepared by simple co-precipitation method. Initially, 2 g of FeCl_3_.4H_2_O and 1 g of FeCl_2_.4H_2_O were dissolved in 50 ml of millipore water and kept in the ultrasonication bath at room temperature for 1 h. 0.1 M urea solution was slowly added drop wise and the sonication was continued for another 15 mins and resultant black precipitate was collected by centrifugation. The supernatant solution was decantated and the final product was dried in vacuum oven to get the black powdered iron oxide nanoparticles.

### 2.4. Encapsulation of dexamethasone on MNP and functionalization with L-carnosine

0.1 g of Fe_3_O_4_ nanoparticles were dissolved in 30 ml of deionized water and placed in the ultrasonication bath for 15 mins to get the homogenous suspension. The PLGA solution was separately prepared by mixing 0.15 g of PLGA in 1:1 ratio of water/acetic acid medium. The two solutions were mixed together and continuously stirred for 2 h to get the PLGA coated iron oxide nanoparticles. Further, it was mixed with 0.1 M of dexamethasone and L-carnosine solution and placed in the sonication bath for 15 mins followed by stirring at 600 rpm and the resulting black precipitate was filtered off and dried at room temperature to get the aligned dm@LMNP composited form.

### 2.5. *In vitro* drug loading and release studies

The in vitro drug loading and release studies of dexamethasone were conducted in phosphate buffer solution at pH 7.4. Different concentrations of the as prepared material such as 100 200, 300, 400, 500 µg was dispersed in PBS solution and 5 ml of Dexamethasone drug solution was added in to them and kept in a shaking incubator at 120 rpm and incubated for 24 h at 37° C in 5% CO_2_. After incubation, the mixture was centrifuged for 5 mins and the supernatant solution was taken for drug loading studies. Again, the supernatant solution was washed with DCM and ethanol water mixture to remove the unreacted compounds. The precipitated nanocomposites were redispersed in fresh PBS medium. The drug encapsulation efficiency and the drug release were determined from the UV-Visible spectrophotometer at 530 nm as reference. The conjugated material was freeze dried and stored at 4° C for further use. The drug releasing studies are also same as that of drug loading and the drug release was tested in different pH like 7.4 and 4.6. The drug loading and incorporated efficiency was calculated using the following equations.

$$\text{Drug}\;\text{loading}\;\%=\frac{\text{Weight}\;\text{of}\;\text{encapsulated}\;\text{drug}\;\text{in}\;\text{nanocomposite}}{{\text{Weight}\;\text{of}\;\text{Fe}}_{3}O_{4}-\text{PLGA}-\text{Dexamethasone}\;\text{nanocomposite}}\times \;100$$

$$\text{Encapsulation efficiency }\%=\frac{\text{Weight of drug in nanocomposite}}{\text{Weight of initial amount of drug}}\times \text{1}00$$

### 2.6. *In vitro* cellular cytotoxicity assay study

Human fibroblast cell line was grown in Dulbecco’s modified eagles medium (DMEM) contains 10% fetal bovine serum (FBS) and 1% penicillin/streptomycin antibiotic which are incubated at 37^0^ C in humidified atmosphere with 95% air and 5% CO_2_. MTT assay was performed to study the cellular cytotoxicity (Sun et al., 2016). In a typical procedure, fibroblast cells having density of 10^4^ per well were put in the flat bottomed 96 well plate and incubated for 24 h at the above conditions. To that, different concentrations of Fe_3_O_4_/PLGA nanoparticles ranging from 100 to 500 µg/mL were added. The zero concentration of the prepared nanoparticle is considered as control experiment. After that, 20 µl of MTT (3-[4, 5 dimethylthiazol-2-yl]-2, 5 diphenyltetrazolium bromide) assay was added to each plate and incubated for 4 h. Then the medium was discarded and the cells were treated with 100 µl of dimethyl sulfoxide solvent. All the experiments were repeated for three times. Absorbance was measured at 570 nm using UV-Vis spectrophotometer. The cell viability was calculated using the formula given below.

$$\text{Cell viability }\%\frac{{\text{OD}}_{\text{T}}-{\text{OD}}_{\text{B}}}{{\text{OD}}_{\text{C}}-{\text{OD}}_{\text{B}}}\times \text{1}00$$

where, OD_T_ and OD_B_ is the optical density of test sample and the plate well without the cells respectively. OD_C_ is the optical density of control.

### 2.7. *In vitro* fluorescence and ROS analyses

For cell viability analyses, Annexin V and 7AAD staining were performed. BMVEC collected from transwells were washed twice with cold PBS, and then resuspended in the provided binding buffer and stained in the dark for 15 min at room temperature with PE Annexin V and 7-AAD. Heating BMVEC at 55 °C for 10 min and vigorous scraping of the cells served as positive controls for apoptosis. The cells were analyzed by flow cytometry within 30 min of staining.

ROS assay was performed using brain capillary endothelial cells (BCECs) (5 × 10^3^ cells/well). The cells were serum starved for 3 h, washed and treated with 50 μM pure drug/nanoparticles and 200 μM of H_2_O_2_ as a positive control. The cells were irradiated with light after 2 h and treated with 1 μM of 2′, 7′-dichlorofluorescindiacetate (DCFH DA) at 37 °C for 10 min and then washed twice to remove excess dye, further incubated with phosphate buffer saline for 1 h. The resultant fluorescence was measured using multi plate reader at an excitation of 495 nm and emission of 529 nm.

Ethidium bromide (EtBr) and Acridine orange (AO) (100 mg/mL) were mixed with cell suspension (1 × 10^5^ cells/mL) and smeared on microscopic cover slips. The fibroplast cells were harvested and washed by Na_2_HPO_4_–KH_2_PO_4_ buffer solution (pH 7.2) and stained (1 mL of AO/EtBr). After incubation, the cells were washed twice with PBS and observed under a fluorescence microscope at a magnification of 40 x with an excitation filter at 480 nm.

### 2.8. Statistical studies

The obtained results were represented as means of ± SD values (*n* = 3). One way ANOVA and Paired *t*-tests were used to analyze the test results with the significance value of *p* < .05.

## 3. Results and discussion

### 3.1. Chemical structure and morphology examinations

**Figure 1.**
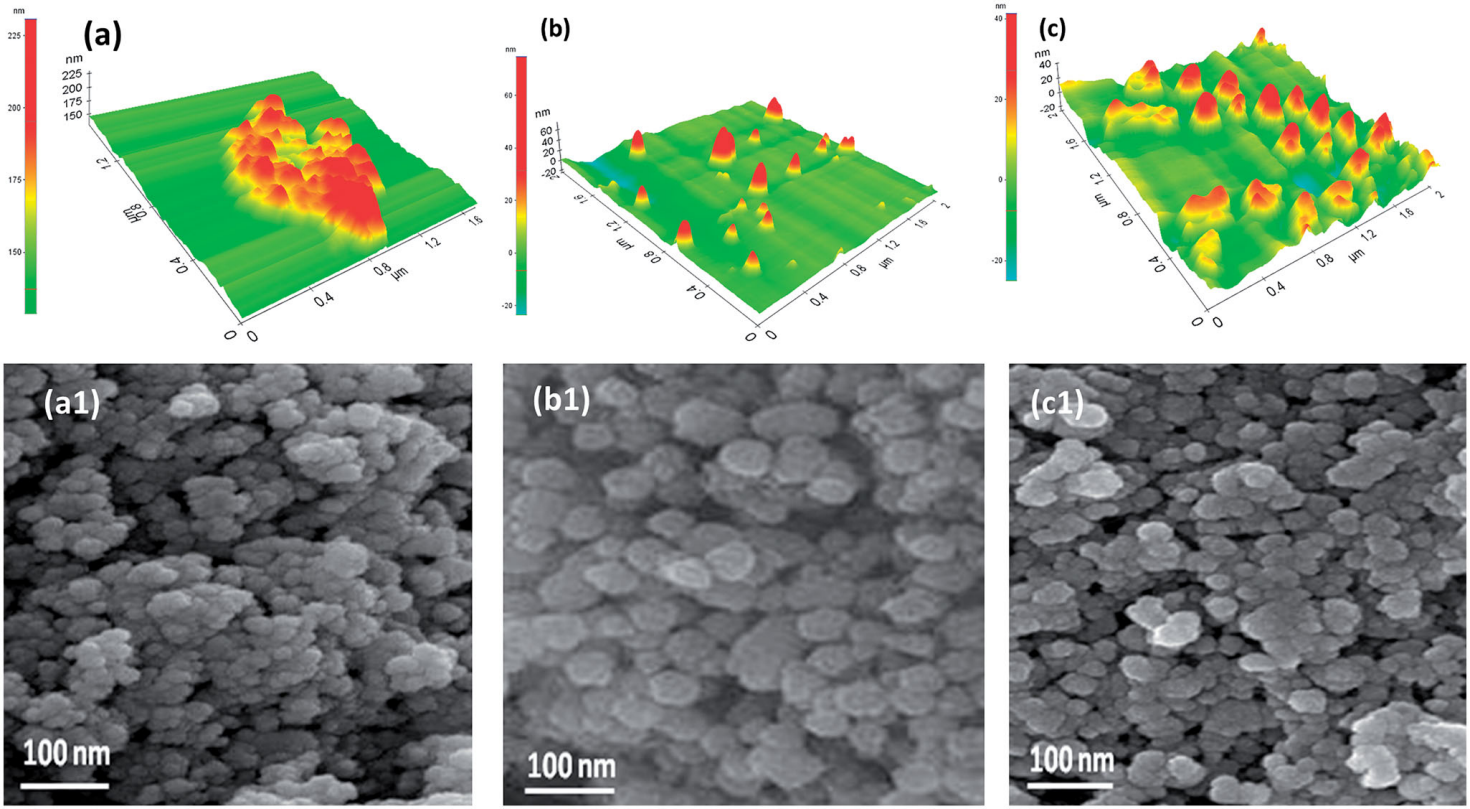
Surface morphology observation through AFM and SEM techniques: (a, a1) Fe_3_O_4_ nanoparticles, (b, b1) MNP composited particles and (c, c1) dm@LMNP nanoformulations.

**Figure 2.**
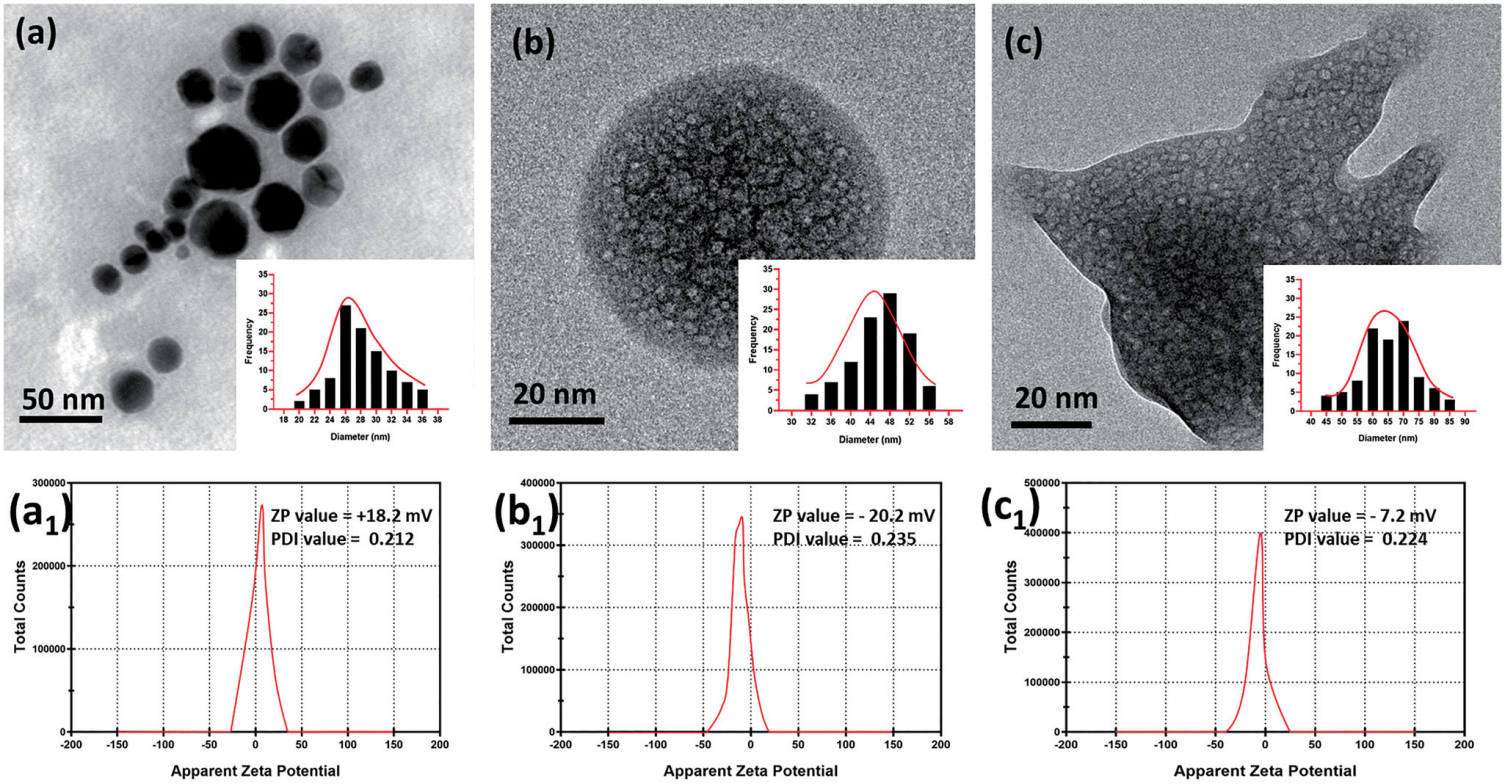
TEM image of (a) Fe_3_O_4_ nanoparticles, (b) MNP composited particles and (c) dm@LMNP nanoformulations (Particles size distribution (PSD) of these synthesized particles have been inserted); The polydispersity index (PDI) and zeta potential value of the prepared bare Fe_3_O_4_ nanoparticles, MNP and dm@LMNP nanoformulations have been labeled as a1, b1 and c1, respectively.

The surface morphology of MN, MNP and dm@LMNP composites were investigated from AFM and SEM microscopic techniques. Figure 1(a,a1) shows the morphology of Fe_3_O_4_ nanoparticles that exhibited as spherical shaped nanoparticles is well agreed with the literature (Yang & Lan, 2014) and in some spots the agglomerated particles are clearly visible which may be due to the high surface tension of the nanoparticles. As shown in Figure 1(b) and b1, upon addition of PLGA, the surface morphology appeared as sphere like particles and decrease in agglomeration was clearly seen indicating the electrostatic repulsion between polymer and the magnetic nanoparticles. After loading of dexamethasone drug, the morphology remained as such and the narrow distribution of particles is clearly seen in Figure 1(c,c1). In order to get further details about the morphology and the particle size TEM analysis was carried out. Figure 2 shows the transmission (TEM) surface morphology of the MNP (Figure 2(a,b)) and dm@LMNP (Figure 2(c)), which is well agreed with SEM pictures and revealed the sphere-shaped particles uniformly distributed without aggregation. The particle size was calculated as 50 nm. In this work, we have combined different polymeric and drug components with iron oxide nanoparticles, hence, the particles size could be varied by the influences of the components. Particles size distribution analysis (Figure 2(a–c)) was observed and demonstrated that MNP and dm@LMNP have average particles size diameter of 64.5 and 47.6, respectively, which exhibits larger particles size when compared to the bare MN (26.4) due to the influences of PLGA and drug molecules presented on iron oxide nanoparticles. As shown in Figure 2(a_1_,b_1_,c_1_), the zeta potential analysis results exhibited that bare MN have positive surface charge of +18.2 mV and PLGA coated MNP and dm@LMNP composited materials have negative surface change of −20.2 and −7.2, respectively, which confirms PLGA coated materials have active carboxylic groups. From the previous reports, the negative surface charge of composited materials could be highly favorable for drug releasing and facilitate to prevent aggregation of nanoparticles from positive charge metal ions (Inbaraj & Chen, 2012).

**Figure 3.**
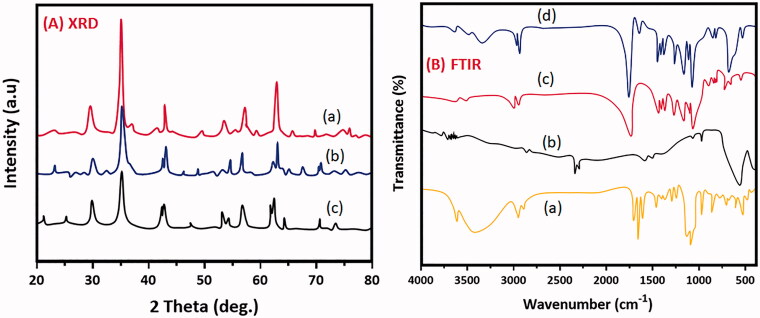
Analysis results of XRD patterns (a) Fe_3_O_4_ nanoparticles, (b) MNP composited particles and (c) dm@LMNP nanoformulations; FTIR spectrums of (a) dexamethasone, (b) Fe_3_O_4_ nanoparticles, (c) MNP and (d) dm@LMNP nanoformulations.

The crystal structure and the diffraction planes were studied from XRD patterns as exhibited in Figure 3(A). The diffraction peaks at 30.1, 35.5, 43.2, 57.3 and 62.8 is well coincide with (220), (311), (400), (511) and (440) and all the crystal planes are due to cubic spinel structured Fe_3_O_4_ nanoparticles (You et al., 2019) and is shown in Figure 3(Aa) which is well agreed with the JCPDS card number. As expected, the addition of PLGA (75/25 grade) with Fe_3_O_4_ does not influence the crystal structure but the encapsulated PLGA was clearly diffracted at 2 ϴ = 170° which revealed the formation of MNP composited form which is shown in Figure 3(Ab). Figure 3(Ac) shows the XRD of dm@LMNP composite does not showed significant difference than that of the earlier. The average crystallize size was calculated using Debye Scherrer equation and is in the range of 10–15 nm. The full width half maximum of (220) plane is 0.3 Å indicating the high crystallinity of the synthesized materials. No other impurity peaks were detected on the entire scan range. The sharp peaks obtained indicating the well crystalline nature of the prepared material and more than two peaks were clearly seen suggesting the polycrystalline nature of the material.

FTIR spectroscopy is a technique used for the effective identification of encapsulated material and to study the bending and stretching vibrations of the polymers and the changes if any occur due to the drug conjugation. Figure 3(B) shows the FTIR spectrum of MN, MNP and dm@LMNP. For magnetic Fe_3_O_4_ nanoparticles, the stretching band at 584.8 cm^−1^ is due to the Fe-O-Fe bond revealed the formation of iron oxide nanoparticles (Shagholani et al., 2015; Basu et al., 2018). A peak at 1632 cm^−1^ is corresponding to the bending vibration of O-H adsorbed on the Fe_3_O_4_ surface which is shown in Figure 3(Ba). On addition of PLGA, all the characteristic peaks were clearly visible at their corresponding wave numbers. The major peaks at 1310, 1452, 1752, and 2996 cm^−1^ are due to the CH (CH)_3_, CH_2_, C = O and C-H respectively proved the existence of PLGA in the nanocomposites and is shown in Figure 3(Bb). As seen in Figure 3(Bc), the dexamethasone bands were not visible and this may be due to the complete encapsulation of the drug in the internal cores of PLGA and most of the bands appeared at similar wave numbers as that of PLGA.

### 3.2. *In vitro* drug release

**Figure 4.**
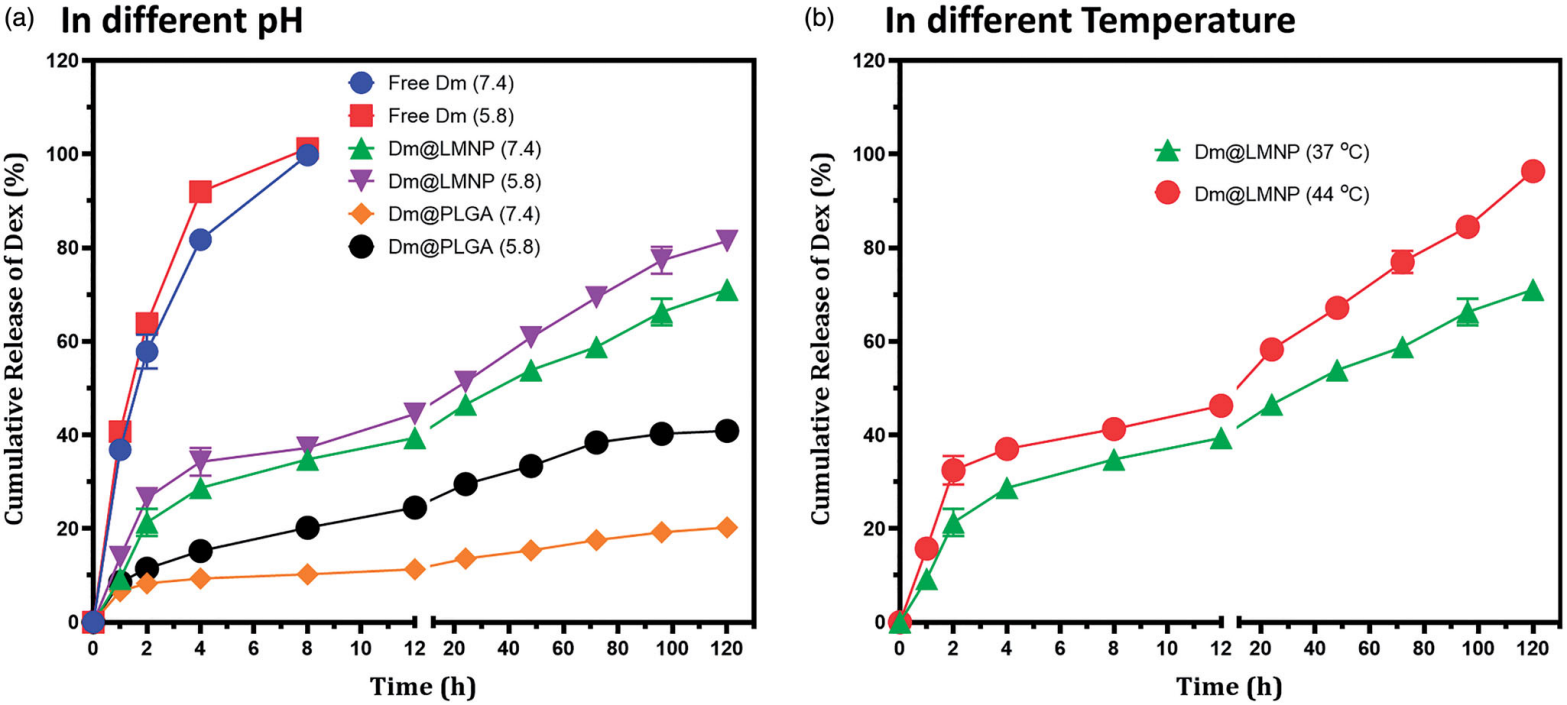
*In vitro* drug (dexamethasone) release profile of dm@LMNP nano-formulation at different physiological pH conditions.

Nanoparticles have high surface to volume ratio and they enable other molecules to incorporate easily. Encapsulation efficiency may depend on the chemical nature and polarity of the drug molecules. Drug loading and encapsulation efficiency was investigated and it was found that 95.6 ± 0.2% of drug was loaded into the LMNP nanoparticles that confirm the suitability of the proposed material as drug carrier for targeted drug delivery applications. The entrapment efficiency was estimated as 74.12% which is higher than the previous reports (Papadimitriou & Bikiaris, 2009; Filippousi et al., 2013). Dexamethasone was taken as model drug for our study and the drug release kinetics was studied using UV spectrophotometer in particular time points in phosphate buffer solution at pH 7.4 and 5.8 at 37 °C. Figure 4 shows the cumulative release of dexamethasone from PLGA nanoparticles and dm@LMNP composite as a function of time. The observations of *in vitro* drug release were demonstrated that free dexamethasone has very quick release as reported in previous reports (Español et al., 2016; Wanawananon et al., 2016). The proposed conjugated dm@LMNP material had displayed triple phase drug release profile and was described as the insignificant discharge was happened in the physiological (pH 7.4 and 5.8) conditions. After 128 h, sustainable discharge of drug was observed from dm@LMNP at pH conditions of 7.4 and 5.8 such as 20.5% and 41.5%, respectively when compared to the dm@PLGA nanoparticles (Figure 4). The initial sustainable and controlled release of drug molecules which may be due to the strong interactions with the nanocomposite surface. The controlled and constant release of the drug might be because of the uniform dispersal into the LMNP composite form and slow degradation of the MNP nanoparticles. The increased drug release was observed at pH 5.8 which was due to the incorporation of L-carnosine and the presence of porous nanostructures. The proposed LMNP nanoparticles demonstrated sustained release and more retention of the dexamethasone drug. It is clear from the observed results (Figure 4(b)) that drug release potential have been improved by influences of increased temperature, due to the enhancing thermal agitation behavior of magnetic nanoparticles, which causes for the detach drug molecules from their surfaces. Those factors have been helped to improve the drug release rate, which reaches about ~ 80% in short time. The observed results exhibited that the higher drug release rate (~ 96%) of dm@LMNP was increased at increased temperature (44 °C), which demonstrated that hyper-thermal magnetic nanoparticle have been influenced the drug release profile. In addition, that, it is difficult to use temperature sensitive drug delivery system in the BBB crossing and ischemic stroke therapies.

### 3.3. *In vitro* cellular uptake

**Figure 5.**
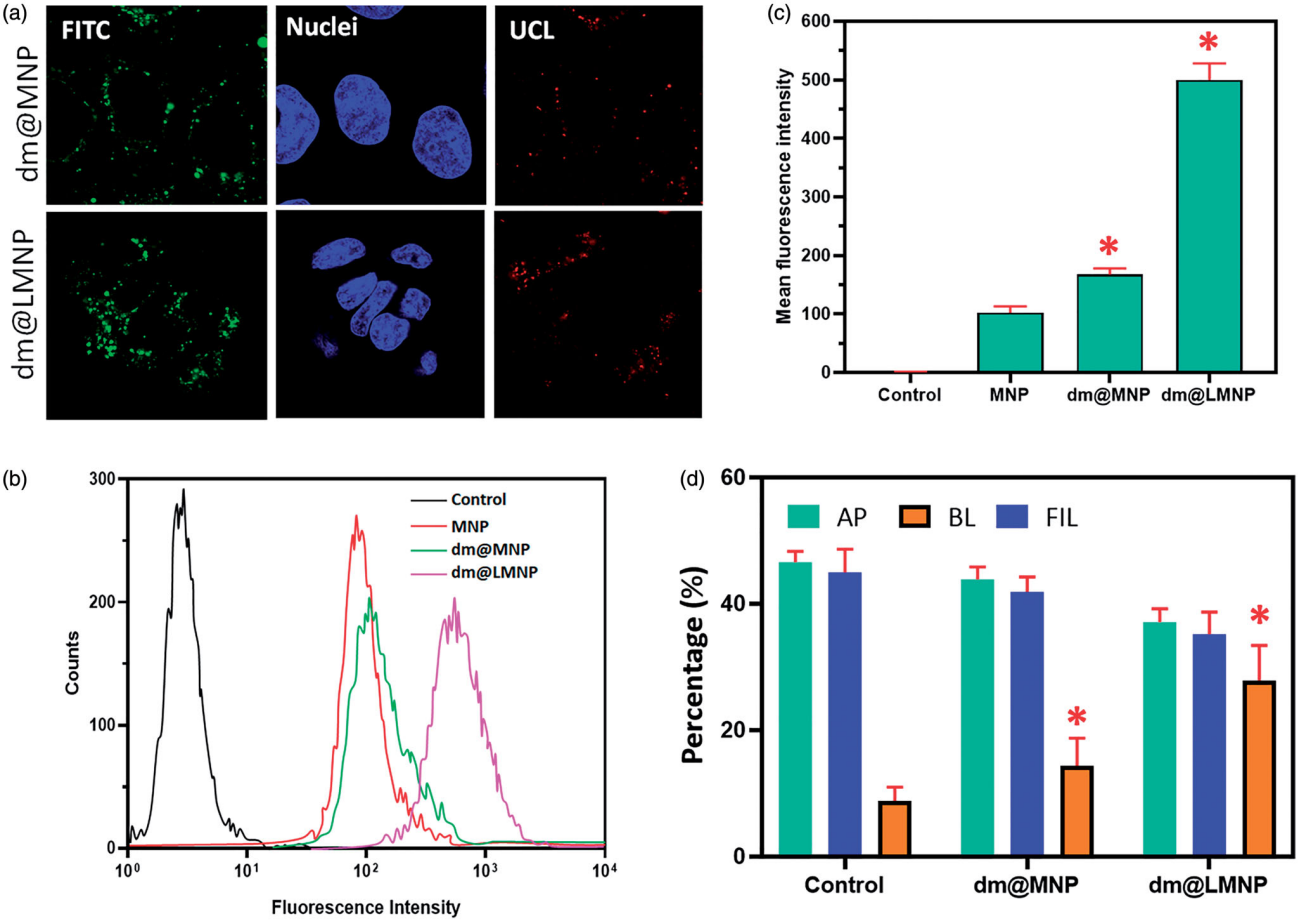
*In vitro* cellular uptake analysis results (a) CLSM observations of BCECs cells incubated with dm@MNP and dm@LMNP formulations; (b & c) flow cytometric analysis results of cellular uptake of dm@LMNP compared with other nanoformulations including control; (d) *In vitro* analysis of BBB crossing of dm@LMNP through transcytosis technique (*n* = 3, *p < .05, error bar indicates SD).

To examine the targeting ability and *in vitro* cellular uptake of the prepared dm@MNP by the brain capillary endothelial (BCECs) cells (Figure 5). The BCEC cells are the chief component of BBB and wisely used for the analysis of brain drug delivery systems in previous reports. The dm@LMNP have greater cellular uptake compared to the dm@MNP material, which was confirmed through the flow cytometry analysis, due to the overexpression’s of LPR by L-carnosine dipeptide ligands. The flow cytometric analysis of dm@LMNP composite form exhibited positive fluorescent signal with treatment of Rhodamine B isothiocyanate (RITC) compared to the dm@MNP composited form at incubation time of 7 h. In addition, the CLSM microscopic images BCECs treated with the prepared nanocomposited particles also confirmed the flow cytometry results and demonstrated the greater cellular uptakes as exhibited in Figure 5(a). The dm@LMNP samples treated BCECs displayed the strong red fluorescence signal around the cell nucleus when compared to the cells treated with dm@MNP nanoparticles at same incubated conditions. The results of fluorescence intensity analysis clearly proved that dm@LMNP have strong cellular uptake compared to the MNPLGA, which can be corresponded to the specific targeting influences of L-carnosine dipeptide to the LRP receptor on the brain cell membranes. Then, the MNPs nanoparticles uptake was quantitatively examined by the inductively coupled plasma optical emission (ICP-OE) spectrometry on the cellular monolayer, apical side and basolateral side after co-incubation of drug loaded nanoparticles over 24 h. The in vitro BBB transwell assay model exposed that ~ 28% of dm@LMNP nanovesicles have successfully crossed into the BBB layer and it was observed on the basolateral side through transwell assay. The sample of dm@MNPs have lower BBB penetration (∼16%) when compared to the dm@LMNP nanovesicles, demonstrating that dm@LMNP have greated targeting and penetrating effect on BBB layer. This observation has been demonstrated that incorporation of dipeptide L-carnosine has played efficient role in BBB crossing transcytosis under LPR receptors as exhibited in Figure 5(d). Hence, we have found no particles charge influences in the particles transportations.

### 3.4. *In vitro* cytotoxicity assay and ROS regenerative ability

**Figure 6.**
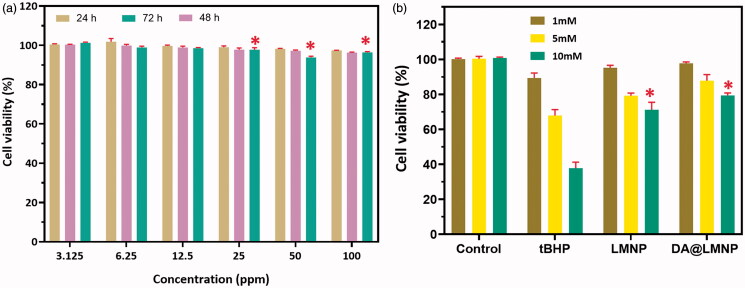
(a) *In vitro* cytotoxicity (MTT-8 assay) of brain capillary endothelial cells (BCECs) cell lines with different concentrations of dm@LMNP for 100 h of exposure; (b) *In vitro* protective ability analysis of the dm@LMNP treated with BCECs induced by tBHP (*n* = 3, *p < .05, error bar indicates SD).

In order to test the biocompatibility of dm@LMNP nanocomposites, MTT assay was performed and its cytotoxicity toward brain capillary endothelial cells (BCECs) was investigated as shown in Figure 6. MTT assay exhibited the toxicity levels in % of cell viability. The BCECs cells treated with PBS was considered as having 100% viability. To test the cell viability, dm@LMNP nanocomposite was incubated with different concentrations such as 3.125, 6.25, 12.5, 25, 50 and 100 ppm/mL with BCECs cell lines which displayed 93.45 to 90.13% cell viability after 120 h confirmed the high cytotoxicity for fibroblast cells and is shown in Figure 5(a). But upon incubation with higher concentration (100 ppm mL^−1^), the cell viability was not decreased below 90%, which is proved that prepared materials have higher compatibility which is due to the saturation of the up taking of the drug. In general, the cytotoxicity of drug vesicles could be obtained may be due to the physical properties of the nanocomposites such as size, morphology and surface charge. Particle size less than 100 nm could easily penetrate the cell membrane to generate ROS can cause cell death. Another important parameter associated with cytotoxicity is the surface charge. The negatively charged particles can have specific interaction with the positively charged cell membrane leads to the cell membrane leakage. As expected, the as prepared material did not reveal any significant toxicity for BCECs cell lines which may be used as drug carrier for the treatment of brain diseases. After that, the protective ability of the prepared nanocomposite with L-carnosine against oxidative-stress influenced cell death under treated with tert-butyl hydroperoxide (tBHP). The cell survival was greatly reduced after treatment of bare tBHP, which is exhibited in the Figure 5(b). Then, the protective ability of L-carnosine functionalized nanocomposites was investigated after treatment of tBHP by using MTT assay, which exhibits no obvious decrease of cell survival rate. The enhanced cell viability was achieved with dm@LMNP nanocomposite compared to the dm@MNP confirms that the influence of bioactive and anti-oxidant efficient L-carnosine dipeptide, which greatly contributed to remove intracellular ROS species.

**Figure 7.**
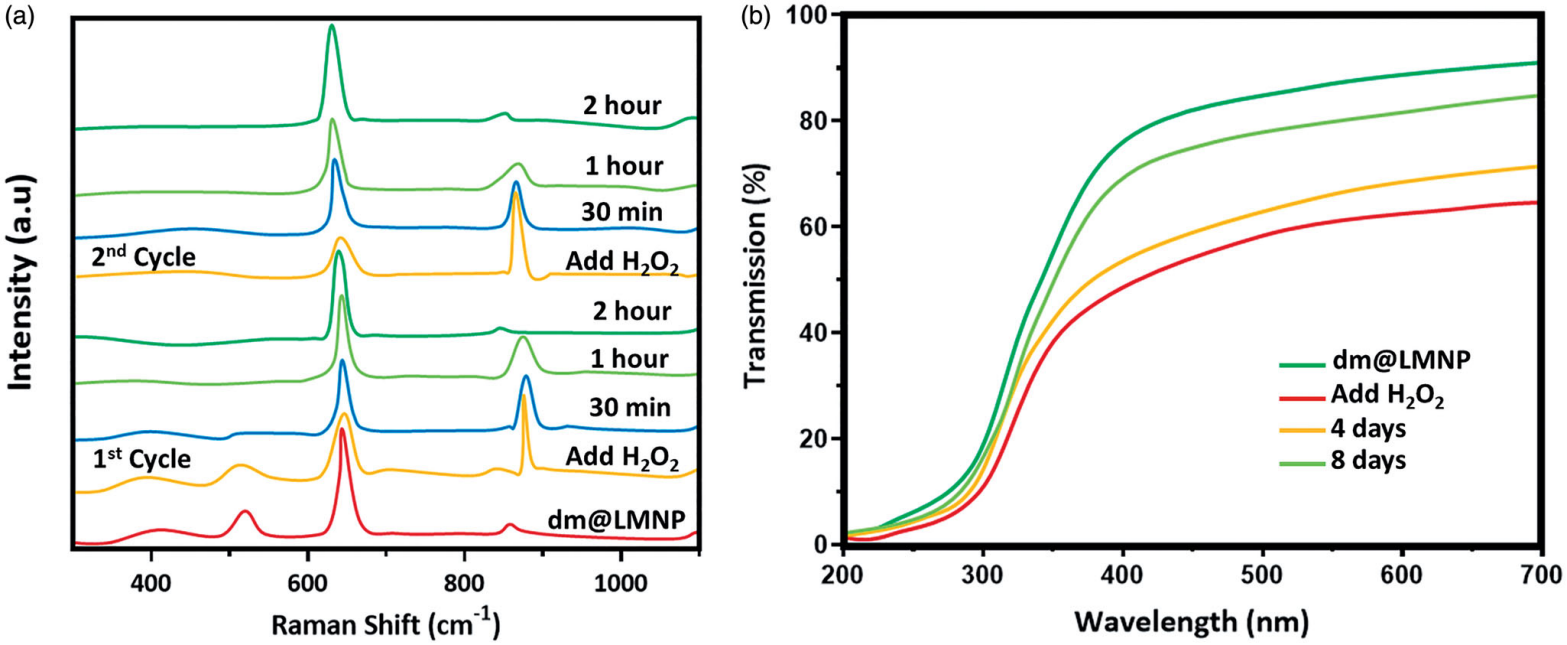
Raman and UV-visible spectroscopic techniques to examine the ROS scavenging ability of the dm@LMNP.

In addition, Raman and UV-visible spectroscopic techniques were used to examine the ROS scavenging ability of the dm@LMNP as exhibited in Figure 7. The radical scavenging ability was determined by using methyl violet and the maximum absorbance of methyl violet was greatly decreased by Fenton reaction due to C = C bond breakage via electrophilic addition of hydroxyl radicals. Though, the absorption degeneration of methyl violet was not affected when added with dm@LMNP nanoformulations, due to the scavenging effect of prepared formulations with peptide molecules. Meanwhile, the auto-regenerative and catalytic effect of the prepared nanoformulations were determined by Raman spectroscopic technique during reaction with H_2_O_2_. The primary peaks of Fe_3_O_4_ nanoparticles can be displayed at 648 cm^−1^, which corresponded to the symmetric mode of oxygen atoms around Fe ions and in order to related to sublattices of oxygen. After immediate reaction with H_2_O_2_, the main peaks were shifted to higher wavenumbers (860 and 890 cm^−1^), while the old peak of cerium oxide was disappeared due to the formation of oxygen-oxygen stretching vibrations of adsorbed peroxide species through deformation of oxygen sublattice of Fe ions. Here, the shifted peaks of peroxide deformations have been changes after appropriate time and return to their original intensities as resembled to an anti-oxidant cycle of prepared formulations. As expected, the adding of H_2_O_2_ again, the regeneration ability of the prepared materials have been efficient and almost indistinguishable to same as first cycle, demonstrating that outstanding recyclable anti-oxidant and catalytic ability of prepared nanoformulations with L-carnosine. It was also verified and confirmed by the transmission UV-vis spectroscopic technique as exhibited Figure 7.

## 4. Conclusions

Dexamethasone loaded MNP nanoparticles functionalized with L-carnosine were successfully prepared and investigated for the BBB crossing and the treatment of ischemic stroke. The prepared nanocomposites were thoroughly characterized using different physiochemical techniques such as SEM, TEM, XRD and FTIR. SEM images showed the formation of spherical shaped nanoparticles without agglomeration with the particle size of 50 nm. The XRD analysis also confirmed the polycrystalline nature of the material and particle size calculated using Debye Scherrer formula is well coincide with the TEM analysis. The FTIR spectrum exhibited the presence of metal oxygen bond and carbon oxygen bond confirmed the formation of Fe_3_O_4_/PLGA composite. The cytotoxicity test was conducted to test their biocompatibility in drug delivery to the brain diseases. The effective blood brain barrier carrier was demonstrated and the drug release study for the ischemic stroke was presented. Based on the above results, it was found that the proposed nanocomposites can act as a suitable drug carrier for ischemic stroke treatment and simultaneous blood brain barrier crossing.
